# Repositioning Antimicrobial Agent Pentamidine as a Disruptor of the Lateral Interactions of Transmembrane Domain 5 of EBV Latent Membrane Protein 1

**DOI:** 10.1371/journal.pone.0047703

**Published:** 2012-10-19

**Authors:** Xiaohui Wang, Zeno Fiorini, Christina Smith, Yingning Zhang, Jing Li, Linda R. Watkins, Hang Yin

**Affiliations:** 1 Department of Chemistry and Biochemistry and BioFrontiers Institute, University of Colorado at Boulder, Boulder, Colorado, United States of America; 2 Department of Psychology and Neuroscience, and the Center for Neuroscience, University of Colorado at Boulder, Boulder, Colorado, United States of America; 3 Department of Rheumatology, Peking Union Medical College Hospital, Chinese Academy of Medical Sciences, Beijing, People’s Republic of China; University of Nebraska - Lincoln, United States of America

## Abstract

The lateral transmembrane protein-protein interactions (PPI) have been regarded as “undruggable” despite their importance in many essential biological processes. The homo-trimerization of transmembrane domain 5 (TMD-5) of latent membrane protein 1 (LMP-1) is critical for the constitutive oncogenic activation of the Epstein-Barr virus (EBV). Herein we repurpose the antimicrobial agent pentamidine as a regulator of LMP-1 TMD-5 lateral interactions. The results of ToxR assay, tryptophan fluorescence assay, courmarin fluorescence dequenching assay, and Bis-Tris sodium dodecyl sulfate polyacrylamide gel electrophoresis (SDS-PAGE) consistently show pentamidine disrupts LMP-1 TMD-5 lateral interactions. Furthermore, pentamidine inhibits LMP-1 signaling, inducing cellular apoptosis and suppressing cell proliferation in the EBV infected B cells. In contrast, EBV negative cells are less susceptible to pentamidine. This study provides a novel non-peptide small molecule agent for regulating LMP-1 TMD-5 lateral interactions.

## Introduction

The Epstein–Barr virus (EBV), one of the world’s most widespread viruses, infects 90–95% of adults in the United States. The infection of EBV in the memory B cells of the adaptive immune system contributes to lymphoid malignancies and lymphoproliferative syndromes in immuno-compromised individuals [1], [2], [3], [4]. Latent membrane protein 1 (LMP-1) is the main oncogenic protein of EBV and is essential for EBV-induced B lymphocyte transformation and immortalization [2], [3], [4]. LMP-1 is an integral membrane protein with six hydrophobic transmembrane helices. Homo-oligomerization of LMP-1′s hydrophobic transmembrane domains (TMD) initializes constitutively active LMP-1 signaling [2], [5]. Recently, the fifth transmembrane domain (TMD-5) of LMP-1 has been identified to mediate LMP-1 oligomerization and signaling [6]. Additionally, a TMD-5 self-association/LMP-1 signaling inhibitor NSC 259242 (Figure S1) has been discovered by cell based screening [7]. However, the NSC 259242’s anti-TMD-5 effect is mild [7]. Pentamidine (Figure S1) is a structural analogue of NSC 259242 and a clinical drug currently used for treatment of protozoa caused infections [8]. In previous structure activity relationship studies [7], the positive charged benzamidine motifs spaced by suitable linker is an essential requirement for the LMP-1 TMD-5 inhibitors [7]. The length of TMD-5 transmembrane segment (F144 to A157) is around 20 Å, and the measurement of pentamidine (19.9 Å) indicates that it approximately matches the length of TMD-5. Therefore, in order to discover a more potent and drug-like TMD-5 disruptor, we tried to investigate the possibility of repositioning of pentamidine as the inhibitor of TMD-5 lateral interactions and LMP-1 signaling. The results of this study show pentamidine disrupts TMD-5 lateral interactions, suppresses LMP-1 signaling NF-κB activation, induces caspase 3/7 over-production, and increases the population of EBV positive B cells undergoing apoptosis and proliferation arrest. Compared to NSC 259242, pentamidine is a more potent TMD-5 self-association disruptor and LMP-1 signaling inhibitor. This study provides a novel example of repositioning a clinical drug as a probe for regulating lateral protein-protein interactions (PPIs) in the TMD region of proteins.

## Materials and Methods

### Cells

HeLa cell line and Ramos cell line were obtained from American Type Culture Collection (ATCC, Rockville, MD, USA). The human B lymphoblastoid cell line 721 (Epstein–Barr virus (EBV)-positive) was first established by Kavathas *et*. *al*. [9] and was kindly provided by Dr. Jennifer M. Martin (University of Colorado at Boulder). Ramos NF-κB reporter cell line was obtained from Invivogen (San Diego, CA, USA). E. *coli.* strain FHK12 was kindly provided by Dr. Dieter Langosh (Technische Universit, München, Germany).

**Figure 1:**
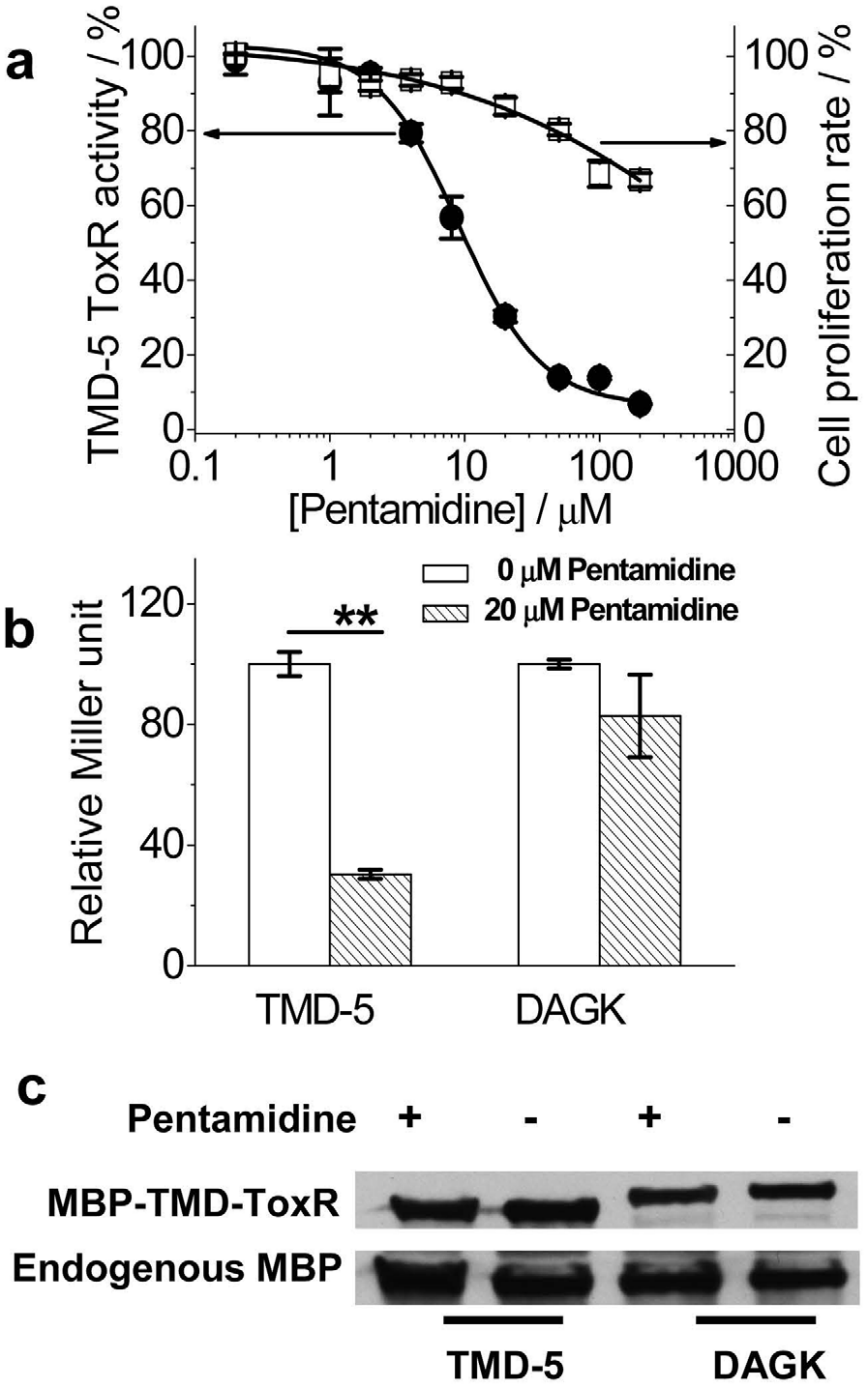
Pentamidine inhibits LMP-1 TMD-5 ToxR activity. (a), Dose-dependent curve of pentamidine inhibiting TMD-5 oligomerization measured by the ToxR assay (IC_50_ = 9.1±0.6 µM). The effect of pentamidine on the *E. coli*. FHK 12 cells proliferation was examined by measuring the absorbance at 600 nm. The Miller unit and the absorbance at 600 nm of the untreated control was set as 100%. (b), ToxR measurement of the effect of pentamidine on the oligomerization of LMP-1 TMD-5 and TMD of diacylglycerol kinase (DAGK). Oligomerization activity in the absence of pentamidine was set as 100%. **p<0.01 by student t-test versus the control. (c), Western blotting showed the chimeric MBP-TMD-ToxR protein expression levels in *E. coli*. FHK 12 cells treated as (b). Endogenous MBP was served as the internal loading control.

### ToxR Assay

pTox7 plasmid was kindly provided by Dr. Dieter Langosh (Technische Universit, München, Germany). The pTox7 plasmid was modified by insertion of a single base (T) after the BamH1 site to keep the proper reading frame for the designed transmembrane sequences [10]. ToxR7-TMD-5 and ToxR7–diacylglycerol kinase (DAGK, which served as the nonspecific control) plasmids were constructed as described previously [10], [11]. ToxR7-TMD-5 plasmid (200 ng) was transformed into 200 µL FHK12 competent cells with heat shock at 42°C for 90 s and incubation on ice for 2 minutes, followed by addition of 800 µL SOC media and incubation with shaking at 37°C for 1 h. 50 µL of the transformation mixture was used to inoculate 5 mL LB + arabinose (0.0025%) and chloramphenicol (30 µg/mL) with different concentrations of pentamidine (Sigma-Aldrich, St. Louis, MO, USA, purity >98% ) in triplicate. Cultures were incubated with shaking at 37°C for 20 h and β-galactosidase activity was measured using a Beckman Coulter DTX 880 plate reader (Beckman Coulter, CA, USA) as described previously [10], [11]. Briefly, 5 µL of culture was transferred to the wells of a Costar 3596 polystyrene 96-well plate (Corning, NY, USA) containing 100 µL Z buffer/chloroform (1% β-mercaptoethanol, 10% chloroform, 89% A buffer: 1 M sodium phosphate, 10 mM KCl, 1 mM MgSO4 and pH 7.0). Cells were lysed by addition of 50 µL Z buffer/SDS (1.6% w/v sodium dodecyl sulfate in Z buffer) and shaking at 28°C for 10 min. 50 µL Z buffer/*o*-nitro phenyl galactoside (ONPG, 0.4% w/v in Z buffer) was added and β-galactosidase activity was measured by monitoring the reaction at 405 nm for a period of 20 min at 28°C. Miller units were calculated using the following equation: Miller units = (OD _405 nm/_min)/OD _600 nm_×1000. The effect of pentamidine on FHK12 *E. coli*. proliferation was also investigated by measuring the OD at 600 nm.

**Figure 2:**
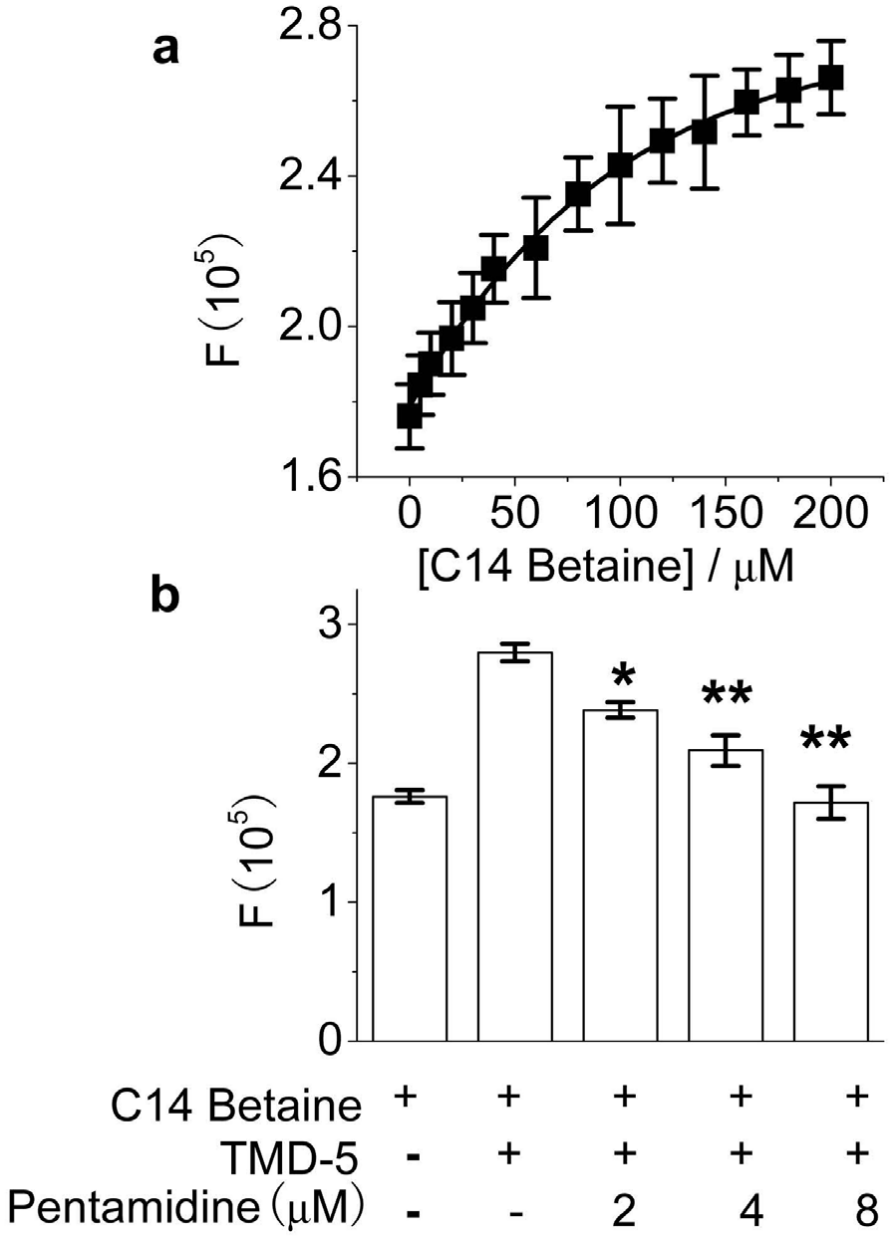
TMD-5 intrinsic Trp fluorescence assay. (a), TMD-5 Trp fluorescence emission intensity increases along increasing C14 betaine micelle concentrations. 1 µM in TMD-5 in 50 mM HEPES (pH = 7.4) buffer was excited by 295 nm and emission at 330 nm was plotted against C14 betaine micelle concentrations. (b), Pentamidine decreases Trp emission fluorescence intensity of TMD-5 in 150 µM C14 betaine and 50 mM HEPES (pH = 7.4) buffer in a dose-dependent manner. *p<0.05 by student t-test versus the fluorescence of TMD-5 in the presence of C14 betaine; **p<0.01 by student t-test versus the fluorescence of TMD-5 in the presence of C14 betaine.

**Figure 3:**
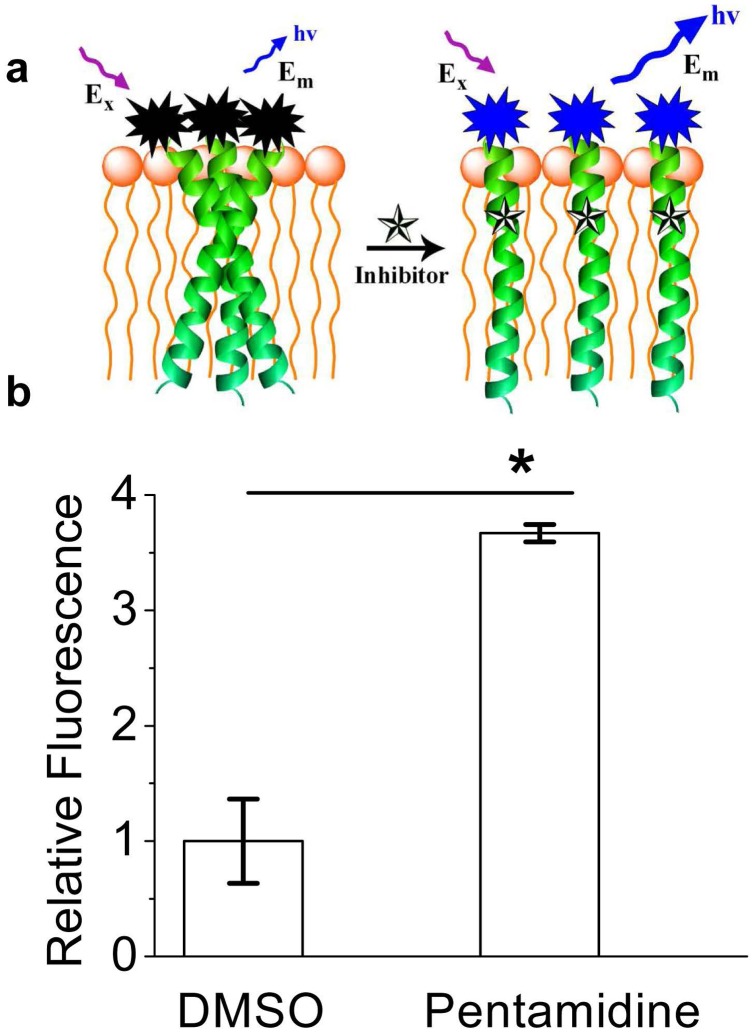
Coumarin fluorescence dequenching assay. (a), Schematic representation of coumarin fluorescence dequenching assay. Coumarin labeled TMD-5 peptide forms trimers in the micelle and coumarin fluorescence is self-quenched. A small molecule that disrupts the TMD-5 oligomerization will suppress the quenching of coumarin fluorescence and a fluorescence increase will be observed. (b), Pentamidine disrupts LMP-1 TMD-5 trimers in the micelle. Pentamidine (64 µM) was added into 100 nM of coumarin labeled TMD-5 solution (50 mM HEPES, pH = 7.4) with 150 µM C14 betaine. Excitation, 360 nm; emission, 430 nm. *p<0.05 by student t-test versus DMSO control.

### Western Blotting

The samples were first separated by 12% sodium dodecyl sulfate polyacrylamide gel electrophoresis (SDS-PAGE) and then electro-blotted to polyvinylidene fluoride (PVDF) membrane. After blocking with 5% bovine serum albumin (BSA), the membranes were incubated with 0.5 µg/mL of maltose binding protein (MBP) primary antibody at room temperature for 2 h. The membranes were washed 5 times in phosphate buffered saline (PBS, 137 mM NaCl, 2.7 mM KCl, 8 mM Na_2_HPO_4_, 1.46 mM KH_2_PO_4,_ pH 7.4) solution with 0.05% of Tween 20 (PBST) for 5 min each and then incubated for 1 h at room temperature with secondary antibody-horseradish peroxidase (HRP) conjugate (50 ng/mL). After extensive washing in PBST, the protein–antibody complexes were visualized by exposure to X-ray film after reacting with Super-Signal West Pico Chemiluminescent Substrate (Pierce, Rockford, IL, USA). FHK12 cells endogenous MBP served as the internal loading control.

**Figure 4:**
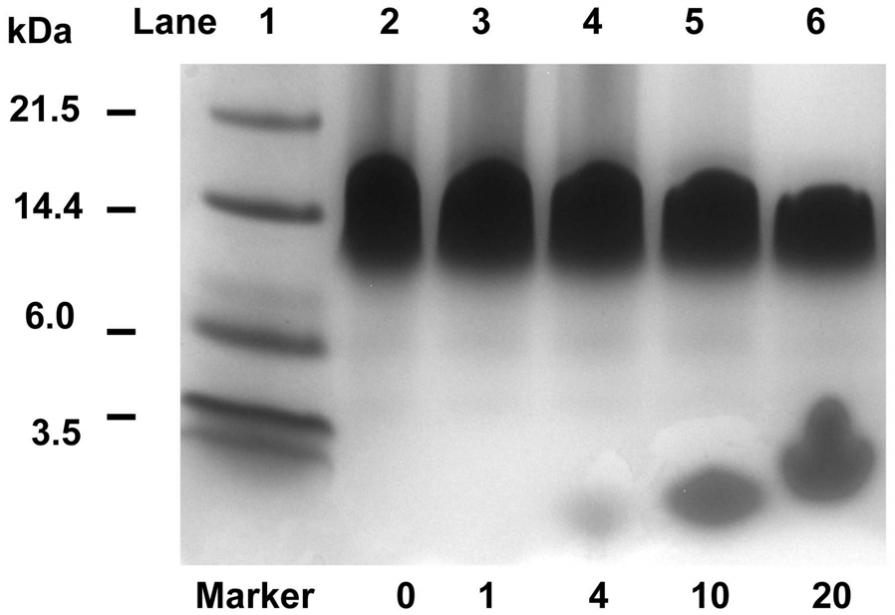
12% Bis-Tris SDS-PAGE analysis of TMD-5. 10 µL of TMD-5 peptide (KKKK-WQLLAFFLAFFLDLILLIIALYL-KKKK) and pentamidine mixture was incubated with equal volume of 2×Laemmli sample buffer at room temperature overnight. The final TMD-5 concentration was 200 µM. The samples were heated at 90°C for 7 min before loading. Electrophoresis was carried out at room temperature with NuPAGE MES SDS running buffer at 150 mV for 45 min. The resulting gel was stained by Coomassie blue staining. The ratios of compound to peptide tested were 0 (lane 2), 1 (lane 3), 4 (lane 4), 10 (lane 5) and 20 (lane 6).

### Peptide Synthesis and Purification

Peptides were synthesized on Rink Amide MBHA resin (100–200 mesh) (Calbiochem-Novabiochem, San Diego, CA, USA) with a substitution level of 0.57 mmol/g. Peptides were synthesized using a CEM Liberty automated synthesizer (CEM, Mathews, NC, USA) with Discovery microwave module at 0.1 mmol scale. Activation of the free amino acids was achieved using HATU (0.40 M solution in N, N-dimethylformamide). The reaction solvent was N-methyl-2-pyrrolidone (HPLC grade, Fisher, PA, USA). Side chain de-protection and simultaneous cleavage from the resin was performed using a mixture of trifluoroacetic acid (TFA)/water/1,2-ethanedithiol/triisopropylsilane (90∶2.5∶2.5∶1,v/v) or trifluoroacetic acid/water/phenol/thioanisole/1,2-ethanedithiol/triisopropylsilane (81.5∶5:5∶5:2.5∶1) at room temperature for two hours. The crude peptides were collected by precipitation with cold (−20°C) diethyl ether (Sigma-Aldrich, St. Louis, MO, USA). The peptides were then purified using Agilent 1200 series semi-preparative reverse phase HPLC system (Santa Clara, CA, USA) with an Agilent Zorbax 300 SB-C8 column using a linear gradient of buffer A (10% 2-propanol, 0.1% trifluoroacetic acid in Millipore water) and buffer B (6∶3:1 2-propanol/acetonitrile/water containing 0.1% trifluoroacetic acid). The identities of the purified peptides were confirmed as the monomeric species by MALDI-TOF mass spectroscopy on a Voyager DE-STR Biospectrometry Workstation (PerSeptive Biosystems, CA, USA).

**Figure 5:**
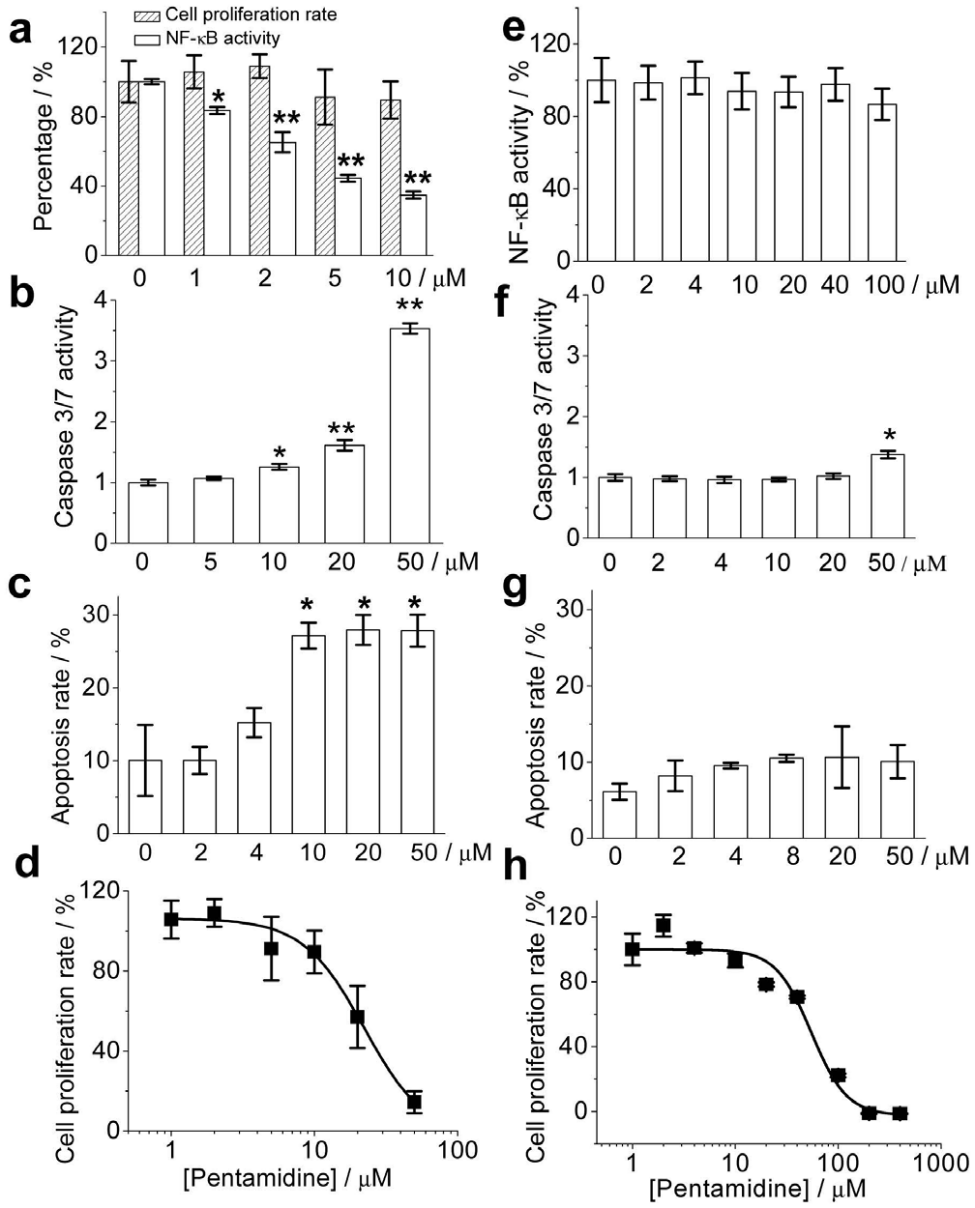
Effect of TMD-5 trimer disruptor pentamidine on NF-κB activity (a, e), caspase3/7 activity (b, f), apoptosis (c, g) and cell proliferation (d, h) in EBV positive B 721 cells (a, b, c, d) and EBV negative B Ramos cells (e, f, g, h). 721 cells/Ramos cells were treated with the indicated concentration of pentamidine for 24 h. The cell proliferation rate was detected by WST-1 assay; constitutive LMP-1 NF-κB activity in 721 cells was determined by Steady-Glo luciferase assay system; NF-κB activity in Ramos cells were determined by SEAP assay; caspase 3/7 activity was detected by the Caspase-Glo® 3/7 assay system; cell apoptosis was detected by AO/EB method. It should be noted that the cell proliferation rate/NF-Κb activity of the untreated cells was normalized as 100% and the caspase 3/7 activity of the untreated control cells was set as 1. *p<0.05 by student t-test versus 0 µM pentamidine group; **p<0.01 by student t-test versus the 0 µM pentamidine group.

Peptides were labeled with coumarin using the following method: 7-Hydroxycoumarin-3-carboxylic acid (100 mg, 0.485 mmol) and HBTU (186 mg, 0.490 mmol) were dissolved in 1∶9 dimethyl sulfoxide/N,Ndimethylformamide and added to the resin. N,N’-Diisopropylethylamine (185 µL, 1.06 mmol) was added and the reaction was stirred for 2–16 h until the Kaiser test indicated that the reaction was complete. Cleavage and purifications were performed as described above.

**Figure 6:**
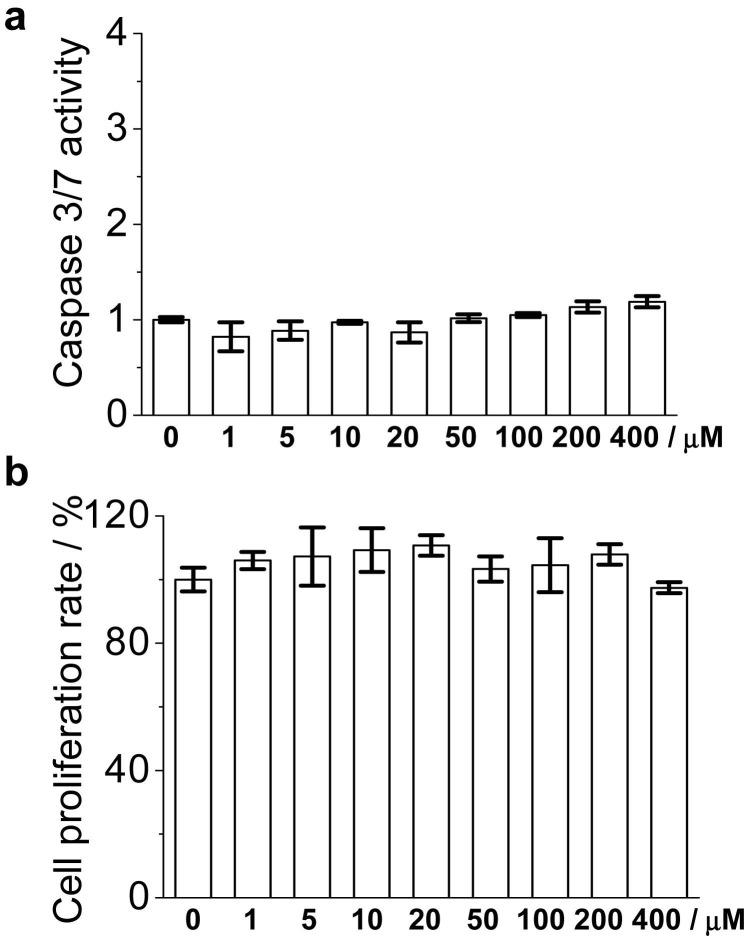
Effect of pentamidine on primary endothelial cell caspase 3/7 activity (a) and cell proliferation (b). Endothelial cells were isolated and cultured as described in Materials and Methods section. Primary endothelial cells were treated with the indicated concentration of pentamidine for 24 h. Caspase 3/7 activity was detected by the Caspase-Glo® 3/7 assay system and the cell proliferation rate was detected by WST-1 assay. It should be noted that the caspase 3/7 activity of the untreated control cells was set as 1 and the cell proliferation rate of the untreated cells was normalized as 100%.

The concentration of TMD-5 peptide (KKKK-WQLLAFFLAFFLDLILLIIALYL-KKKK) derived from LMP-1 was determined by absorbance at 280 nm using an extinction coefficient of 6990 M^−1 ^cm^−1^. The concentration of coumarin labeled TMD-5 peptide (coumarin -GGGPG-WQLLAFFLAFFLDLILLIIALYL-GPGG) was determined by absorbance at 400 nm using an extinction coefficient of 39300 M^−1^ cm^−1^.

### Tryptophan Fluorescence

LMP-1 TMD-5 peptide insertion into micelles was assayed by monitoring changes in the emission intensity of tryptophan as described previously [12]. In order to further increase the solubility of LMP-1 TMD-5, KKKK-TMD-5-KKKK (KKKK-WQLLAFFLAFFLDLILLIIALYL-KKKK) was used. An aliquot of peptide from a concentrated stock was added to 50 mM HEPES buffer (pH 7.4), to a final concentration of 1 µM and was allowed to equilibrate for 5 min at which point the Trp emission spectra was recorded by using 295 nm as the excitation wavelength and 330 nm as the emission wavelength. The sample was then titrated with aliquots of C14 betaine (3-(*N,N-*dimethylmyristylammonio)propanesulfonate, Sigma-Aldrich, St. Louis, MO, USA) micelle. After incubation for 10 min, the Trp emission spectra were re-recorded.

### Coumarin Fluorescence Enhancement Assay

Coumarin labeled TMD-5 peptide was dissolved in trifluoroethanol (TFE) with C14 betaine. The organic solvent was removed with nitrogen under reduced pressure to generate a thin film of peptide/detergent mixture, which was then dissolved in 50 mM HEPES (pH = 7.4) buffer. 100 µL of coumarin labeled TMD-5 peptide (100 nM) solution (50 mM HEPES, 150 µM C14 betaine, pH = 7.4) with the indicated concentration of pentamidine was pipetted into black 96-well plates in triplicate. Samples were mixed and allowed to sit at room temperature in the dark overnight to reach equilibrium and then excited at 360 nm and emission was read at 430 nm using a Beckman Coulter DTX 880 Multimode Detector plate reader. It should be noted that pentamidine has no fluorescence signal at the tested conditions. Appropriate controls were subtracted from the observed coumarin fluorescence signal to eliminate the possible interference from drug fluorescence.

### Bis-Tris Gel Electrophoresis

Electrophoresis was carried out using precast SDS−PAGE gels according to manufacturer’s instructions (12% NuPAGE 10-well Bis-Tris gels, Invitrogen, Grand Island, NY, USA). Briefly, 10 µL of TMD-5 peptide and pentamidine mixture was incubated with equal volume of 2×Laemmli sample buffer (126 mM Tris-HCl, 20% glycerol, 4% SDS, 0.02% bromphenol blue, pH 6.8) at room temperature overnight (TMD-5 final concentration was 200 µM). The ratio of compound to peptide varied from 0 to 20. The samples were heated at 90°C for 7 min before loading. Electrophoresis was carried out at room temperature with NuPAGE MES SDS running buffer (Invitrogen, Grand Island, NY, USA) at 150 mV for 45 min. The resulting gel was stained by Coomassie blue staining as described previously [13], [14].

### B 721 Cell NF-κB Assay

The human B lymphoblastoid cell line 721 was cultured in RPMI medium supplemented with 10% fetal bovine serum (FBS), penicillin (50 unit/mL) and streptomycin (50 µg/mL). NF-κB reporter cells were constructed by Cignal Lenti NF-κB Reporter kit (SABiosciences, MD, USA) and selected by puromycin treatment according to manufacturer’s instructions. The firefly luciferase gene was placed under the control the NF-κB transcriptional response element. Briefly 50 µL of 721 cells (105 cells/mL) were added to the wells of 96-well plate. 50 µL of NF-κB Reporter letivirus suspension (SABiosciences, MD, USA) was added. 8 µg/mL of SureENTRY Transduction Reagent (SABiosciences, MD, USA) was also added to increase the transduction efficiency. After further 48 h incubation, 4 µg/mL puromycin was added to select transduced cells.

B lymphoblastoid 721 NF-κB reporter cells were seeded at a density of 1×104 cells/well in 96-well plates (100 µL/each well). After overnight incubation, different concentrations of pentamidine were added to the cells. Following 24 h of treatment, the NF-κB activity was detected by Steady-Glo Luciferase Assay System (Promega, Madison, MI, USA) according to manufacturer’s instructions. Briefly, 75 µL Steady-Glo Luciferase Assay reagent was added to each well and incubated at room temperature for 15 min. The luminescence was measured by Beckman Coulter DTX880 reader (Beckman Coulter, CA, USA).

### Ramos Cell NF-κB Assay

Ramos NF-κB reporter cell, a B lymphocyte cell line that stably expresses NF-κB-inducible SEAP (secreted embryonic alkaline phosphate) reporter gene, was cultured in supplemented Iscove’s Modified Dulbecco’s medium (IMDM; 10% FBS, 50 unit/mL penicillin, 50 µg/mL streptomycin, 100 µg/mL normocin and 100 mg/mL Zeocin). Cells were seeded at the concentration of 1×10^5^ cells/mL. After overnight incubation, different concentrations of pentamidine were added to the cells. Following 24 h of treatment, the NF-κB activity was detected by Phospha-Light™ SEAP Reporter Gene Assay System (AppliedBiosystems, Foster, CA, USA.), according to manufacturer’s instructions. Briefly, 15 µL supernatant from each well was diluted in 45 µL of 1× dilution buffer, transferred to 96-well plates (Thermo, Walthma, MA, USA), and heated at 65°C for 30 min, then cooled on ice to room temperature. Assay buffer (50 µL/well) was added and, 5 min later, reaction buffer (50 µL/well) is added and allowed to incubate for 20 min at room temperature. The luminescence was measured by Beckman Coulter DTX880 reader (Beckman Coulter, CA, USA).

### Central Nervous System Endothelial Cell Isolation and Culture

Primary cultures of central nervous system (CNS) endothelial cell (EC) were isolated from adult rat brain and spinal cord tissue, as described previously [15], [16], [17]. This method yields cultures that are >98% pure, which was confirmed with positive immunostaining for von Willebrand factor, negative immunostaining for markers of fibroblasts (prolyl 4-hydroxylase) and astrocytes (glial fibrillary acidic protein), and visual inspection of the cells, which had the typical spindle-shaped morphology of CNS EC and formed confluent monolayers that were longitudinally aligned and non-overlapping, as described previously [15]. Rats were anesthetized with isoflurane then decapitated. The brain was dissected out of the skull and the spinal cord was removed by hydraulic extrusion with ice cold physiological saline. The tissue was then processed as follows (all procedures performed using sterile technique): the tissue was placed in an enzymatic digestion solution, containing collagenase type II (Invitrogen, Carlsbad, CA, USA) and DNase I (Sigma-Aldrich, St. Louis, MO, USA) in Hank’s Balanced Salt Solution (HBSS) with calcium and magnesium, then chopped finely with a sterile scalpel. The tissue pieces were triturated in the collagenase II solution with a 25 mL pipette, then incubated at 37°C for 40 min. Following the incubation, the mixture was mixed thoroughly with a 10 mL pipette then a 5 mL pipette until there were no discernible pieces of tissue left. The mixture was centrifuged for removal of the supernatant, and the pellet was then centrifuged in a solution of 20% bovine serum albumin in DMEM. This allowed removal of neurons and glia, leaving behind a pellet of microvessels. The microvessels were then placed in an enzymatic digestion solution, containing collagenase/dispase (Roche, Indianapolis, IN, USA) and DNase I in HBSS without calcium or magnesium, and incubated at 37°C for 30 min. The mixture was then centrifuged, and after removal of the supernatant, the pellet was centrifuged through a 33% Percoll gradient (Amersham Biosciences, Piscataway, NJ, USA) to purify the microvessel fragments. The microvessel fragments were washed once with DMEM then suspended in a basal medium (DMEM/F-12 containing 100 unit/mL penicillin-streptomycin, 50 μg/mL gentamicin, 2 mM GlutaMAX-I, 20% fetal bovine serum, and 1 ng/mL basic fibroblast growth factor (Sigma-Aldrich, St. Louis, MO, USA), supplemented with 4 µg/mL puromycin (a translation inhibitor) which selectively kills microvessel cell types other than EC, due to high expression of the efflux pump P-glycoprotein selectively on EC [18], [19]. This method does not alter EC viability [15]. Microvessel fragments from the CNS tissues were pooled and seeded onto 10 cm petri dishes (coated with a mixture of extracellular matrix proteins including fibronectin, collagen type IV, and collagen type I, then maintained (37°C, 5% CO_2_) for 48 hr in DMEM/F-12 with puromycin, followed by DMEM/F-12 without puromycin, until the EC reached confluence (1 week post-isolation).The cells were then passaged onto 96-well microplates (microplates were again coated with the mixture of extracellular matrix proteins as above) with density of 20 000 cells per well. The cells were used for caspase 3/7 activity measurement and cell proliferation assay experiments after further 24 h incubation.

### Caspase 3/7 Activity Measurement

Cells were treated various concentration of pentamidine. Following 24 h treatment, caspase3/7 activity was determined by Caspase-Glo® 3/7 Assay Systems (Promega, Madison, MI, USA) according to manufacturer’s instruction. Briefly, 100 µL of Caspase-Glo® 3/7 Reagent was added to each well and incubated at room temperature for 1 h. The luminescence of each sample was measured by a Beckman Coulter DTX 880 microplate reader.

### Apoptosis Assay

Cells were treated various concentration of pentamidine. Following 24 h treatment, cells were collected and washed with PBS. Cell concentration was adjusted to 2×10^6^−5×10^6^ cells/mL. 1 µL of ethidiumbromide (EB, 100 µg/mL)/acridine orange (AO, 100 µg/mL) dye mix was added in 10 µl cell suspension, followed by 10 minutes of incubation in the dark [20]. Stained cell suspensions were placed on a clean microscope slide and covered with a cover-slip. Pictures of stained cells were taken on a TE2000-S microscope (Nikon, Tokyo, Japan) equipped with MetaMorph image analysis software (Molecular Devices, Sunnyvale, CA). For AO/EB staining, the live cells have a normal green nucleus; early apoptosis cells have a bright green nucleus with condensed or fragmented chromatin; late apoptosis cells display condensed and fragmented orange chromatin [20]. A minimum of 200 total cells from at least three random microscope fields for each condition were counted and the percentage of cells undergoing apoptosis was determined accordingly.

### Cell Proliferation Assay

Following drug stimulation, 20 µL of Cell Proliferation Reagent WST-1 (Roche Diagnostics GmbH, Mannheim, Germany) was added. After further incubation at 37°C for 1–2 h, the absorbance at 450 nm was measured on a Beckman Coulter DTX 880 microplate reader and 620 nm was chosen as the reference wavelength. The A _450 nm_-A _620 nm_ for the control group was set as 100%.

## Results and Discussion

In order to study the interactions of transmembrane sequences in natural phospholipid bilayers, the Engleman lab developed an *E*.*coli* based ToxR assay [21]. The transmembrane domain of interest is expressed as a chimeric protein with maltose binding protein (MBP) for localization to the periplasm and ToxR to provide a reporter for the level of oligomerization (Figure S2). TMD association causes oligomerization of ToxR, activating the transcription and production of the reporter protein, ß-galactosidase, which can be visualized by the colorimetric reaction of substrate *o*-nitrophenyl galactoside (ONPG). If small molecule inhibitors disrupt the TMD association, decreased ß-galactosidase activity will be observed. As shown in the Figure 1a, pentamidine inhibited TMD-5 ToxR activity in a dose-dependent manner with an IC_50_ of 9.1±0.6 µM. It is about 10-fold more potent than our recently identified TMD-5 disruptor NSC 259242 (IC_50_ = 90.0±10.5 µM) [7]. The effect of pentamidine on *E*.*coli*. FHK 12 cell proliferation was also monitored. Pentamidine showed no significant toxicity up to a concentration of 50 µM. This result eliminated the possibility that the observed pentamidine inhibition of TMD-5 ToxR activity was due to its toxicity. To demonstrate the specificity of pentamidine, we tested its effect on the association of TMD of diacylglycerol kinase (DAGK), a well studied integral membrane protein. As shown in Figure 1b, pentamidine, did not inhibit DAGK TMD oligomerization. The effect of pentamidine on MBP-TMD-ToxR chimeric protein expression was also investigated by western blotting (Figure 1c). Pentamidine apparently did not affect the TMD chimeric protein expression, which eliminated the possibility that the observed lower TMD-5 ToxR activity in the presence of pentamidine was due to it inhibiting TMD-5 chimeric protein expression.

As shown in the Figure 2a, the inserting of TMD-5 into the micelles was investigated by measurement of the fluorescence of the Trp residue in the TMD-5 peptide. The maximum fluorescence intensity increased (Figure 2a), as the peptide inserted into the micelles and formed a homotrimeric complex [6]. As shown in Figure 2b, pentamidine decreased the Trp emission fluorescence intensity of TMD-5 in 150 µM C14 betaine in a dose-dependent manner, suggesting that pentamidine interacts with TMD-5. In order to see whether pentamidine disrupts TMD-5 self-associations, a fluorescence enhancement assay (Figure 3a) was performed to test whether TMD-5 is the direct target of pentamidine. When added to micelles, coumarin labeled TMD-5 peptide forms homotrimer, resulting in the self-quenching of coumarin fluorescence [6]. Upon addition of pentamidine, fluorescence enhancement was observed (Figure 3b). This result indicates pentamidine is a TMD-5 self- association disruptor.

SDS-PAGE is widely used as a membrane mimetic gel to study TMD oligomerization [6], [7], [22]. To further demonstrate that pentamidine disrupts LMP-1 TMD-5 trimer, Bis-Tris SDS-PAGE was performed. As shown in the Figure 4, TMD-5 (KKKK-WQLLAFFLAFFLDLILLIIALYL-KKKK) formed trimers in the membrane mimetic gel, which is consistent with our previous results [6], [7] and shows that the –KKKK linker does not interfere TMD-5 inserting into the membrane and forming trimers. With an increasing ratio of pentamidine to TMD-5, TMD-5 trimer band intensity decreased, and the TMD-5 monomer (∼3.8 kDa) appeared with increased intensity. This result unambiguously shows pentamidine disrupts LMP-1 TMD-5 lateral interactions.

TMD-5 trimerization is essential for LMP-1 constitutive activation. LMP-1 acts as a CD40 mimic by interacting with intracellular signaling proteins and activating the downstream mediator in NF-κB pathway. This signal regulates B cell proliferation, differentiation, and survival [2], [6]. In order to see whether the TMD-5 disruptor pentamidine could inhibit LMP-1 signaling, an NF-κB signaling assay was performed. Naive B cells are the target of EBV infection *in-vivo,* and thus an immortalized EBV positive B 721 cell line was selected for investigating the effect of pentamidine on LMP-1 signaling. As shown in Figure 5a, treatment with pentamidine suppresses constitutive NF-κB activation in EBV positive 721 cells, with an IC_50_ of 4.4±0.6 µM.

NF-κB suppresses cell apoptosis by inducing TNF receptor associated factors (TRAFs) and c-IAP1 and c-IAP2 to suppress caspase-8 activation [23], [24]. Caspases-8 directly processes the pro-caspase3/7 and induces early apoptosis marker caspase3/7 expression. To test whether LMP-1 NF-κB signaling inhibitor pentamidine can induce transformed B 721 cells apoptosis, the effect of pentamidine on caspase3/7 over-expression was first investigated. As shown in Figure 5b, pentamidine induced pro-apoptotic caspase3/7 over-expression, causing the EBV positive B cells to undergo apoptosis in a dose-dependent manner (Figure 5c). However, the correlation of caspase 3/7 expression level and the extent of apoptosis was not observed, which had been also documented by previous reports [25], [26]. Inhibition of positive B cells proliferation with an IC_50_ of 23.4±6.1 µM was observed (Figure 5d). The pentamidine concentration resulting in total cell growth inhibition (TGI) in 721 cells was 22.6 µM (Figure S3), lower than the average (43.1 µM) in TGI of EBV negative NCI-60 cells, a panel of 60 diverse human cancer cell lines representing leukemia, melanoma, and cancers of the lung, colon, brain, ovary, breast, prostate, and kidney [27], [28]. This result indicates pentamidine is more sensitive to EBV positive 721 than EBV negative cells.

To further test the specificity of pentamidine on EBV signaling, the effects of pentamidine on EBV negative B Ramos cells were investigated. As shown in Figure 5e, pentamidine did not inhibit the basal NF-κB activity of Ramos cells, even at the concentration of 100 µM. Compared to EBV positive 721 cells, Ramos cells were less susceptible to pentamidine induced caspase 3/7 overproduction (Figure 5f) and apoptosis (Figure 5g, Figure S4). The IC_50_ of pentamidine inhibiting Ramos cell proliferation was 58.3±7.6 µM (Figure 5h), statistically higher than that of 721 cells (23.4±6.1 µM). HeLa cell line is EBV negative and it was also less susceptible to pentamidine induced caspase 3/7 overproduction (Figure S5a) and apoptosis (Figure S5b). Furthermore, the effect of pentamidine on primary cell (EBV negative) was also investigated. Primary central nervous system (CNS) endothelial cells were isolated from rats and cultured. Pentamidine did not induce caspase 3/7 overproduction (Figure 6a) and had no apparent cell proliferation inhibition (Figure 6b) toward primary endothelial cells, even the concentration of 400 µM. Collectively, the results demonstrate that pentamidine inhibits LMP-1 signaling, induces cellular apoptosis, and suppresses cell proliferation in the EBV infected B cells. Contrastly, pentamidine is less sensitive to EBV negative cells.

## Conclusions

Pentamidine is a clinical drug for treatment of protozoa caused infection, however, its exact mechanism is unknown. Herein, we repurposed the antimicrobial medication pentamidine as a regulator of LMP-1 TMD-5 lateral interactions. Pentamidine disrupts TMD-5 trimerization and self-association. Furthermore, pentamidine specifically inhibits EBV LMP-1 signaling. Significant progress has been made in the development of small molecule regulators of PPIs [29], [30], [31], however, the development of small molecular probes to interrogate protein transmembrane domains is still lagging behind [32], [33], [34], [35]. Herein we provide a novel non-peptide small molecule agent for regulating LMP-1 TMD-5 lateral interactions. Compared to a peptidomimetic, a small molecule anti-TMD agent is more resistant to degradation [36]. This kind of small molecule agent is also superior to antibody-based methods, which can not be used in cellular membranes [32]. Additionally, pentamidine is an FDA approved drug since 1989, which eliminates the possibility of severe toxicology. Due to these advantages, the anti-TMD-5 small molecule pentamidine is inherently powerful as a molecular probe for interrogating LMP-1 TMD-5 lateral interactions in cellular membranes. EBV infection is nearly ubiquitous among adults, can lead to infectious mononucleosis and malignant lymphomas, and increases risk of autoimmune diseases [2], [37], [38]. Antimicrobial medication pentamidine may be repurposed as an essential therapeutic for preventing, diagnosing, and treating EBV caused diseases.

## Supporting Information

Figure S1 Structure of NSC 259242 and pentamidine.
(TIF)

Figure S2 Cartoon schematic representation of the ToxR assay. The LMP-1 TMD-5 is expressed as a chimeric protein with maltose binding protein (MBP) for location to the periplasm and ToxR to provide a report of the level of oligomerization. TMD-induced oligomerization causes oligomerization of ToxR, activation of transcription and production of the reporter protein, ß-galactosidase, which can be visualized by color reaction of substrate o-nitrophenyl galactoside (ONPG). If small molecule disrupts the TMD association, decreased ß-galactosidase activity will be observed.
(TIF)

Figure S3 Comparison of pentamidine concentration resulting in total cell growth inhibition (TGI) in EBV positive B 721 cell and EBV negative NCI-60 cells. TGI of 721 cell was determined according to the method described by US National Cancer Institute (NCI) Developmental Therapeutics Program (DTP) [27], [28]. The NCI-60, a panel of 60 diverse human cancer cell lines representing leukemia, melanoma, and cancers of the lung, colon, brain, ovary, breast, prostate, and kidney. All the NCI-60 cell lines are EBV negative. The TGIs of pentamidine (NSC No. 620107) on NCI-60 were obtained from NCI DTP (http://dtp.nci.nih.gov/dtpstandard/servlet/MeanGraph?searchtype=NSC&searchlist=620107&outputformat=HTML&outputmedium=page&chemnameboolean=AND&debugswitch=false&assaytype=&testshortname=NCICancerScreenCurrentData&dataarraylength=55&endpt=TGI&button=MeanGraph&highconc=-4.0) and data are represented by mean ± standard error of the mean.
(TIF)

Figure S4 AO/EB staining of B cells treated with pentamidine (50 µM, 24 h). (a), representative image of EBV positive 721 cell treated with DMSO control; (b), representative image of EBV positive 721 cell treated with pentamidine; (c), representative image of EBV negative Ramos cell treated with DMSO control; (d), representative image of EBV negative Ramos cell treated with pentamidine. For AO/EB staining, the live cells have a normal green nucleus; early apoptosis cells have a bright green nucleus with condensed or fragmented chromatin; late apoptosis cells display condensed and fragmented orange chromatin.
(TIF)

Figure S5 Effect of pentamidine on HeLa cells. (a), effect of pentamidine on HeLa cell caspase3/7 activity. (b)**,** effect of pentamidine on HeLa cell proliferation rate. An IC_50_ = 44.1±5.9 µM was derived. HeLa cells were cultured in DMEM medium supplemented 10% FBS, penicillin (50 unit/mL) and streptomycin (50 µg/mL). Cells were seeded at 96 well plate with a density of 10 000 cells per well. After overnight incubation, various concentrations of pentamidine were added. After 24 h treatment, WST-1 assay and caspase 3/7 assay were performed as described in Materials and Methods section.
(TIF)
