# Supplementary figures and images for: Repositioning Antimicrobial Agent Pentamidine as a Disruptor of the Lateral Interactions of Transmembrane Domain 5 of EBV Latent Membrane Protein 1
Source: PLoS One. 2012 Oct 19;7(10):e47703. doi: 10.1371/journal.pone.0047703 (PMC3477141; doi:10.1371/journal.pone.0047703)

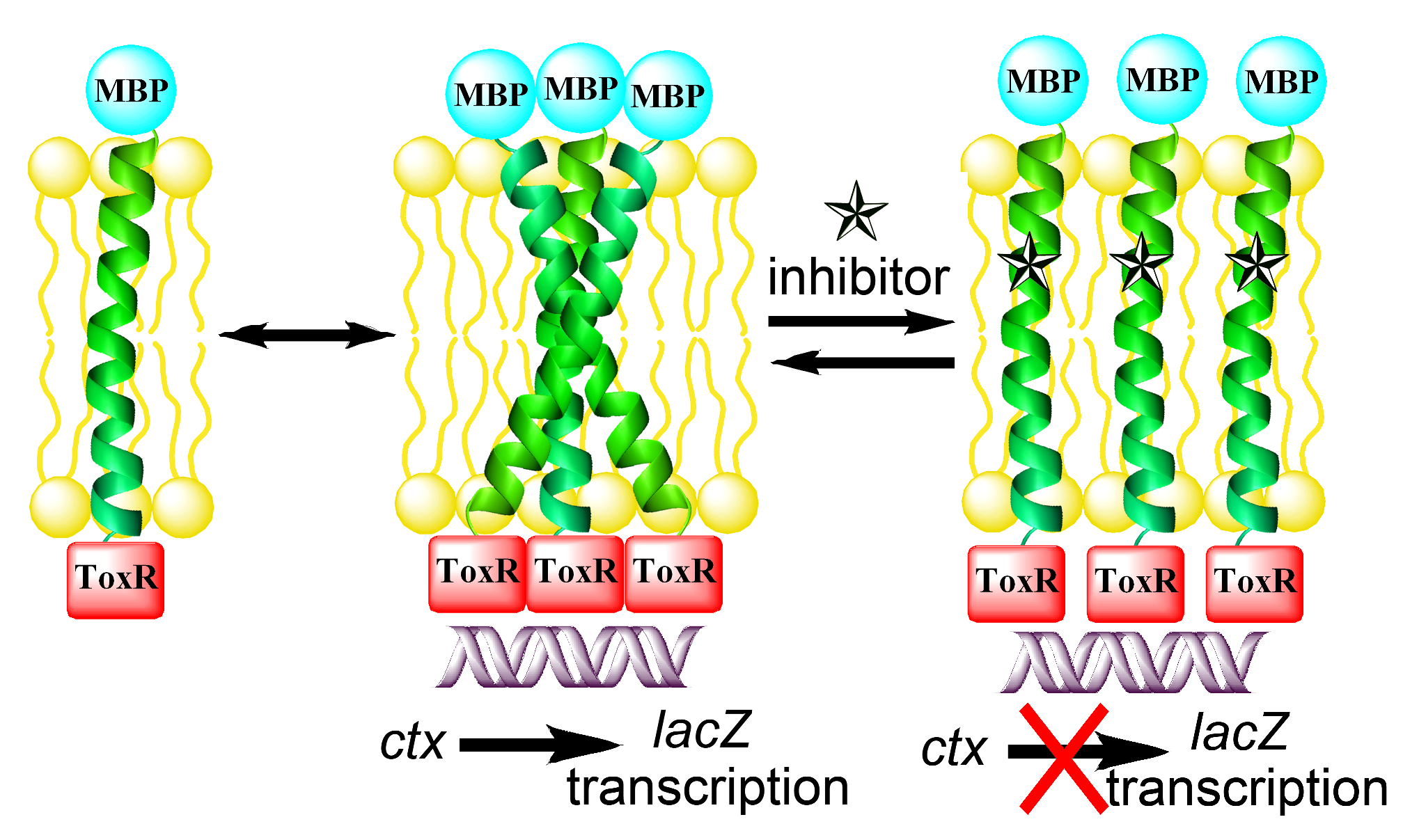

Supplement: Figure S2 — Cartoon schematic representation of the ToxR assay. The LMP-1 TMD-5 is expressed as a chimeric protein with maltose binding protein (MBP) for location to the periplasm and ToxR to provide a report of the level of oligomerization. TMD-induced oligomerization causes oligomerization of ToxR, activation of transcription and production of the reporter protein, ß-galactosidase, which can be visualized by color reaction of substrate o-nitrophenyl galactoside (ONPG). If small molecule disrupts the TMD association, decreased ß-galactosidase activity will be observed. (TIF) [file pone.0047703.s002.tif]

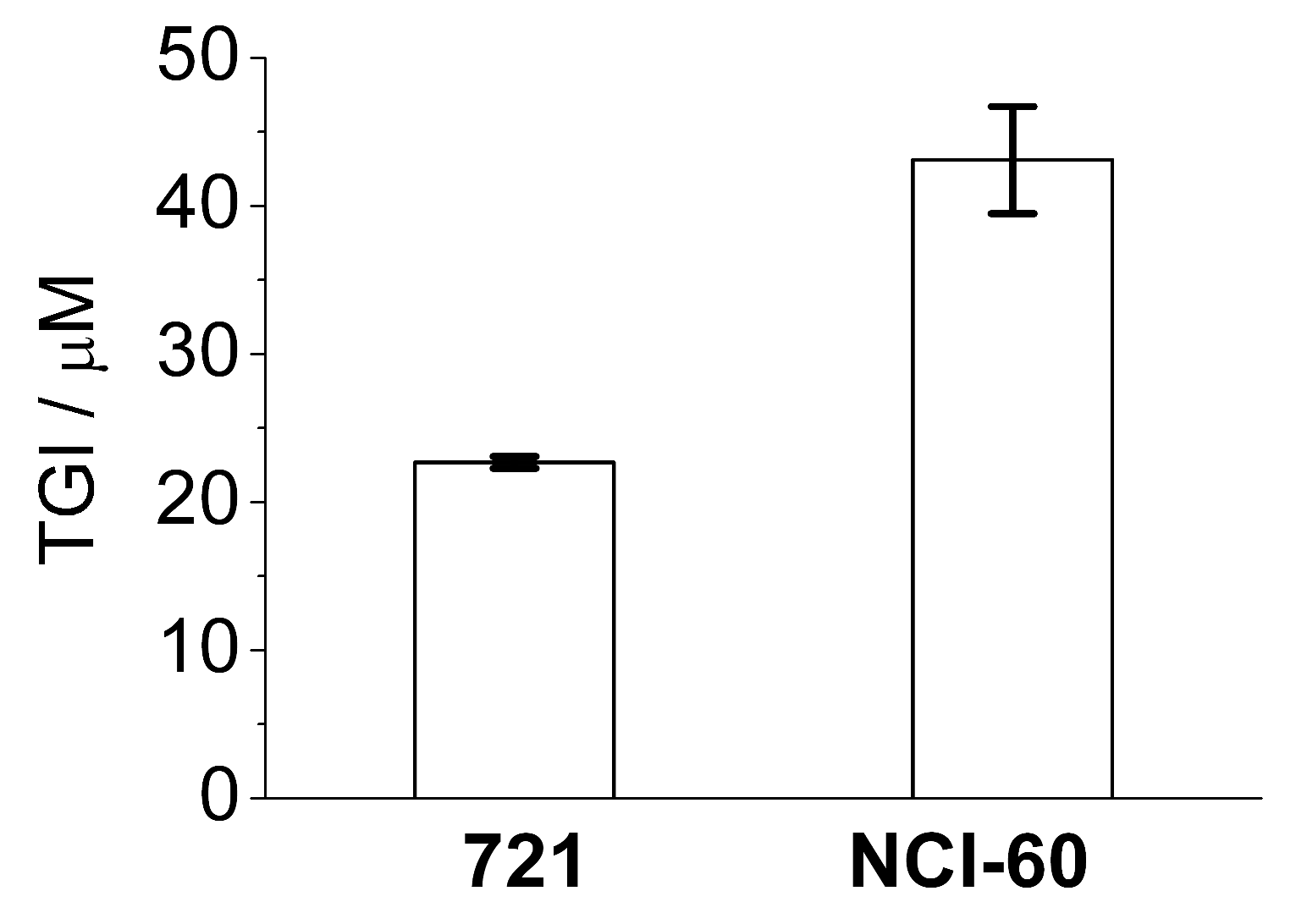

Supplement: Figure S3 — Comparison of pentamidine concentration resulting in total cell growth inhibition (TGI) in EBV positive B 721 cell and EBV negative NCI-60 cells. TGI of 721 cell was determined according to the method described by US National Cancer Institute (NCI) Developmental Therapeutics Program (DTP) [27], [28]. The NCI-60, a panel of 60 diverse human cancer cell lines representing leukemia, melanoma, and cancers of the lung, colon, brain, ovary, breast, prostate, and kidney. All the NCI-60 cell lines are EBV negative. The TGIs of pentamidine (NSC No. 620107) on NCI-60 were obtained from NCI DTP (http://dtp.nci.nih.gov/dtpstandard/servlet/MeanGraph?searchtype=NSC&searchlist=620107&outputformat=HTML&outputmedium=page&chemnameboolean=AND&debugswitch=false&assaytype=&testshortname=NCICancerScreenCurrentData&dataarraylength=55&endpt=TGI&button=MeanGraph&highconc=-4.0) and data are represented by mean ± standard error of the mean. (TIF) [file pone.0047703.s003.tif]

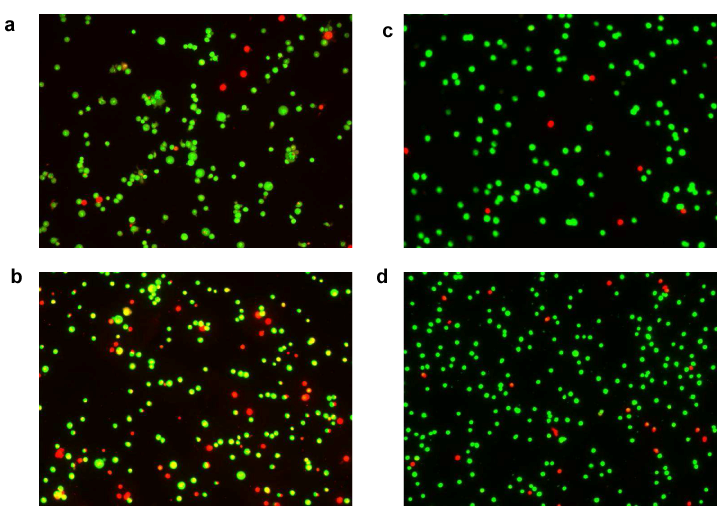

Supplement: Figure S4 — AO/EB staining of B cells treated with pentamidine (50 µM, 24 h). (a), representative image of EBV positive 721 cell treated with DMSO control; (b), representative image of EBV positive 721 cell treated with pentamidine; (c), representative image of EBV negative Ramos cell treated with DMSO control; (d), representative image of EBV negative Ramos cell treated with pentamidine. For AO/EB staining, the live cells have a normal green nucleus; early apoptosis cells have a bright green nucleus with condensed or fragmented chromatin; late apoptosis cells display condensed and fragmented orange chromatin. (TIF) [file pone.0047703.s004.tif]

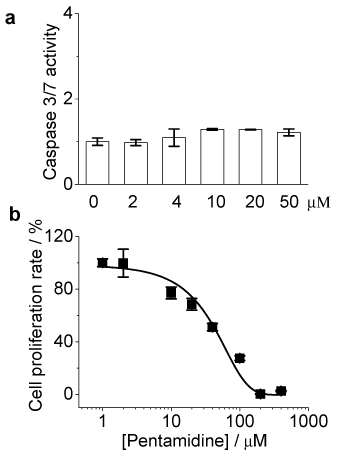

Supplement: Figure S5 — Effect of pentamidine on HeLa cells. (a), effect of pentamidine on HeLa cell caspase3/7 activity. (b), effect of pentamidine on HeLa cell proliferation rate. An IC50 = 44.1±5.9 µM was derived. HeLa cells were cultured in DMEM medium supplemented 10% FBS, penicillin (50 unit/mL) and streptomycin (50 µg/mL). Cells were seeded at 96 well plate with a density of 10 000 cells per well. After overnight incubation, various concentrations of pentamidine were added. After 24 h treatment, WST-1 assay and caspase 3/7 assay were performed as described in Materials and Methods section. (TIF) [file pone.0047703.s005.tif]
